# Characterization of URB Series Synthetic Cannabinoids by HRMS and UHPLC–MS/MS

**DOI:** 10.3390/ph16020201

**Published:** 2023-01-29

**Authors:** Marco Agostini, Donata Favretto, Caterina Renzoni, Susanna Vogliardi, Andrea Duranti

**Affiliations:** 1 Laboratory of Toxicology AST1, Via Lombroso 15, 61122 Pesaro, Italy; 2 Legal Medicine and Toxicology, University Hospital of Padova, Via Falloppio 50, 35121 Padova, Italy; 3 Department of Pharmaceutical Sciences, Via Marzolo 5, 35121 Padova, Italy; 4 Department of Biomolecular Sciences, University of Urbino Carlo Bo, Piazza del Rinascimento 6, 61029 Urbino, Italy

**Keywords:** NPS, synthetic cannabinoids, URB compounds, HRMS, UHPLC–MS/MS, toxicology

## Abstract

A large number of synthetic cannabinoids are included in new psychoactive substances (NPS) and constitute an open research area in analytical pharmaceutical and toxicology when methods are needed to unambiguously identify these substances and their metabolites in biological fluids. A full molecular characterization of five synthetic molecules of the URB series that is able to interact with the endocannabinoid system was achieved with a high-resolution mass spectrometry (HRMS) in positive ion electrospray ionization and collisional experiments on the protonated parent ions, obtaining characteristic fragmentation patterns. Ultra-high-performance liquid chromatography coupled with a triple quadrupole (UHPLC-MS/MS) has also been used, which can help develop methods for screening and confirming synthetic cannabinoids in biological fluids.

## 1. Introduction

Cannabis contains more than 500 compounds, of which at least 100 have pharmacological properties [1] and are therefore classified as phytocannabinoids [2,3]. It is currently the most widely used illicit drug in Europe, with around 22.2 million European adults reporting its use in 2021 [4]. In recent decades, studies on the endocannabinoid system (ECS) have commanded great interest, above all due to the implications deriving from cannabinoid (CB) receptors, which were discovered in the 1990s and are involved in many physiological and pharmacological events [5,6,7,8,9,10]. CB_1_ receptors are mainly present in some parts of the central nervous system (CNS), (predominantly the cerebellum, basal ganglia, hippocampus, and cerebral cortex), while CB_2_ are mainly found in the immune system (primarily B cells and natural killers). Despite this, the CB_1_s are present, albeit scarcely, in districts other than the CNS [8] and the CB_2_s centrally [11], respectively. For more detail on the CB_1_ and CB_2_ receptors, see [9,12]. When CB receptors are activated, there is a decrease in the concentrations of cyclic adenosine monophosphate (cAMP), a messenger that is essential for regulating many cellular functions. In fact, following the binding with suitable ligands, they determine an agonist or antagonist activity, depending on whether they are stimulated or blocked. On the other hand, they can also determine a modulating action through the inhibition of the enzymatic proteins responsible for the metabolism of the endogenous molecules that physiologically bind to them [10]. Indeed, the latter, among which the most studied are *N*-arachidonoylethanolamine (anandamide, AEA) [13] and 2-arachidonoyl-*sn*-glycerol (2-AG) [14,15], plays a very important role in the regulation of many physiological processes after being released on demand from phospholipid membranes [16,17]. The basic concept is that endocannabinoids are produced (and consequently act) when and where it is needed. On the contrary, the administration of non-endogenous molecules that bind to the CB receptors [18,19], in particular when the action is aimed at the CNS, can cause effects of various kinds, such as those exerted by the main psychoactive drug of the cannabis, that is ∆^9^–tetrahydrocannabinol (THC) [20], or those carried out by non-psychotropic cannabidiol (CBD) [21], which have antipodal pharmacological effects despite their similar structural characteristics. ECS is therefore to be considered as a reference system for the discovery of new drugs.

Evidence of the importance of substances capable of interfering with CB receptors is represented by the presence on the market of medicines containing active pharmaceutical ingredients that are able to bind to CB receptors, such as Cesamet^®^ (nabilone), prescribed for the improvement of chemotherapy-induced nausea and vomiting (CINV) states in patients who are not responding to conventional antiemetic therapies. Marinol^®^ (dronabinol), administered for CINV and appetite stimulation in patients with AIDS (acquired immune deficiency syndrome), and Sativex^®^ (THC and cannabidiol), used for the symptomatic relief of the pain and/or the management of the neuropathic pain and spasticity in adults with multiple sclerosis and not responsive to other antispasticity therapies or other marketed drugs. More recently, a new drug containing cannabidiol (Epidiolex^®^) has been approved by the Food and Drug Administration for the treatment of seizures associated with two rare and severe forms of epilepsy, that is, Lennox–Gastaut syndrome and Dravet syndrome, in patients who are two years of age and older [2,21,22,23].

On the other hand, direct and indiscriminate activation of the ECs receptors, particularly of the central CB_1_, can cause several side effects, such as dry mouth, muscle weakness, myalgia, palpitations, blurred vision, memory impairment, disorientation, hallucinations, and paranoia [24,25,26,27]. This problem is accentuated in all situations in which there is an uncontrolled intake that leads to abuse and involves substances of both natural and synthetic origin.

In 2008, a synthetic cannabinoid, namely JWH-018 [28], was first identified in Europe as a constituent of the herbal products available on the web, and was advertised as incense, air fragrances, and herbal blends not intended for human consumption [29]. These products were sold under fanciful trade names (Spice, K2), and quickly spread to several countries as they could be consumed alternatively to cannabis because they were not detectable by the classic drug tests in urine. This situation has allowed the marketing of products able to induce similar or more powerful effects than cannabis without incurring penalties because they are not known and are not listed among the prohibited substances [30]. Since then, the number of “spice” products and new synthetic cannabinoids has steadily increased, so much so that, very recently, 280 individual synthetic cannabinoid receptor agonists (SCRAs) have been reported to the United Nations Office on Drugs and Crime (UNODC) Early Warnings Advisory from 90 different countries up to 2019 [31], and others have been identified in recent years. SCRAs are part of the large family of the so-called new psychoactive substances (NPS), to which, according to UNODC, all substances of abuse belong, either in a pure form or as a preparation, which are not controlled by the 1961 Single Convention on Narcotic Drugs or the 1971 Convention on Psychotropic Substances, but which may pose a public health threat [32]. Among the main classes of NPS, we can include, in addition to SCRAs, synthetic opioids, stimulants, psychedelics, hallucinogens, and so on [33]. Moreover, NPS from 133 countries and territories have been reported [34]. Consequently, the increase in NPS [33,34,35,36] and smart drugs has led to restrictive and monitoring actions by the institutions involved. The continuous emergence of NPS on the market, however, makes legislative restrictions inadequate and the monitoring task of prevention agencies more difficult.

The recent appearance and widespread diffusion of legal hemp-based products (resin, leaves, and inflorescences) with a low THC content but adulterated with highly active synthetic cannabinoids has made the scenario even more complex. In fact, industrial hemp with a low THC concentration is cheaper and the addition of a small amounts of synthetic cannabinoids is enough to mimic the indiscriminate activity aimed at CB targets. This dangerous way of spreading synthetic cannabinoids puts people’s health at risk, not only of those who knowingly buy the drug but also of those who are convinced that they are buying a harmless natural product [4].

At present, most of the cannabimimetic substances identified in seizures of illegal materials in Europe and in other countries derive from commercial products already known, with various abbreviations (e.g., AM, AB, CP, JWH, HU, RCS, UR, URB, and WIN), and belonging to different chemical classes (e.g., adamantoylindoles, aminoalkylindoles, benzoylindoles, cyclohexylphenols, dibenzopyrans, naphthoylindoles, naphthylmethylindoles, naphthylmethylindenes, phenylacylindoles, tetramethylcyclopropylketoindoles, indazole carboxamides, and naphthoylpyrroles) [37]. Recently, an interesting study aiming to explore the steric requirements at CB_1_ and CB_2_ receptors and the role of the related amino acid side chain has been developed that uses some SCRAs [38].

The continuous introduction of new analogues of existing CB drugs is certainly a legal problem, but it is also a major cause of analytical and toxicological uncertainty [39]. In fact, the lack of certified reference standards and the inadequacy of knowledge relating to biological activity and metabolism make it difficult to determine and evaluate new compounds, especially biological matrices. Therefore, the increase in knowledge and the characterization of new CB compounds would be important for their identification. 

**Figure 1:**
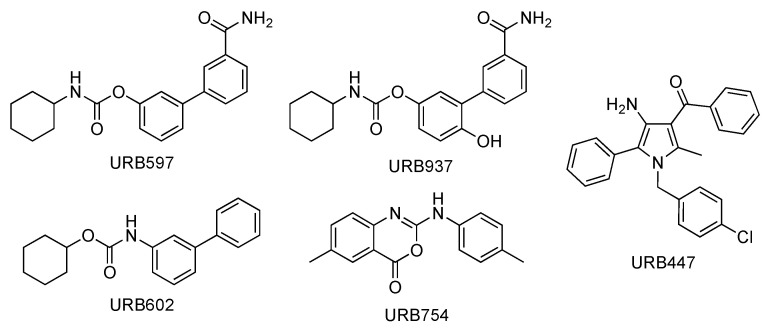
Chemical structures of URB compounds.

In this work, a full molecular characterization by high-resolution mass spectrometry (HRMS) and ultra-high-performance liquid chromatography (UHPLC)–MS/MS of five synthetic molecules of the URB series designed to interact indirectly or directly with ECS (Figure 1) is presented for the first time.

The molecules presented in Figure 1 are defined as synthetic cannabinoids under the broader definition of molecules interfering with the endocannabinoid system without exerting a direct action on the CB receptors (a more classical definition). On the other hand, the EMCDDA (European Monitoring Center for Drugs and Drug Addiction) also includes synthetic cannabinoids molecules that do not bind the receptor.

The compounds of the URB series studied here are representative of molecules able to interfere indirectly (enzyme inhibitors) or directly (ligands) with the biological targets of the endocannabinoid system. These include the following molecules: (A) Two carbamates that inhibit fatty acid amide hydrolase (FAAH), which catalyze the intracellular hydrolysis of the endocannabinoid AEA, and are able to exert anxiolytic and analgesic properties (cyclohexylcarbamic acid 3′-carbamoylbiphenyl-3-yl ester, URB597) [40] or restricted peripheral analgesic effects (cyclohexylcarbamic acid 3′-carbamoyl-6-hydroxybiphenyl-3-yl ester, URB937) [41]. They play a very important role in the panorama of FAAH inhibitors because they were the first ever with a good overall profile. Still today, they constitute reference molecules in the specific research oriented toward the study of molecules that are able to interfere with the endocannabinoid system. So much of this is true that URB597 is the most widely used pharmacological tool in the field, the use of which serves to further understand pharmacological applications. Both are covalent and irreversible inhibitors. (B) A reverse carbamic analogue of FAAH inhibitors that inactivates the enzyme degrading 2-AG, which is a monoglyceride lipase (MGL), and is able to relieve the stress-induced pain and induce neuroprotection (biphenyl-3-yl carbamic acid cyclohexyl ester, URB602 [42]). It is a covalent and partially reversible inhibitor. (C) A benzoxazinone derivative initially studied as an MGL inhibitor (6-methyl-2-*p*-tolylaminobenzo[*d*]oxazin-4-one, URB754) [43,44]. (D) Finally, a peripherally restricted mixed CB_1_ antagonist/CB_2_ agonist pyrrolic drug endowed with anti-obesity, neuroprotective, and antitumor properties [45,46], and an interesting anti-inflammatory profile (data not available) {[4-amino-1-(4-chlorobenzyl)-2-methyl-5-phenyl-1*H*-pyrrol-3-yl] (phenyl)methanone, URB447. These compounds were included among the monitored commercial substances. Notably, URB597 was found in herbal products in both Japan [47] and the United States of America [48], which was notified by the European Union Early Warning System in 2013 (found in Poland) [49], while URB754 has been reported in the literature as a new designer drug [50]. 

Although, at the moment, the phenomena of abuse and fatality have not been correlated with the considered drugs, the molecular characterization and MS fragmentation behavior of the protonated molecules reported here are important for the unambiguous identification of the substances. They also play a key role in the future development of LC–MS/MS or LC–HRMS screening methods that are useful for their research on seized samples.

In fact, the substances studied in this work are potentially dangerous because they are active at very low concentrations (lower than most direct cannabinoids). Furthermore, they are emerging drugs and sufficient epidemiological data to describe their consumption have not yet been acquired (on the other hand, if no one includes them in the screening of real intoxication cases, no one can estimate their prevalence).

## 2. Results and Discussion

**Figure 2:**
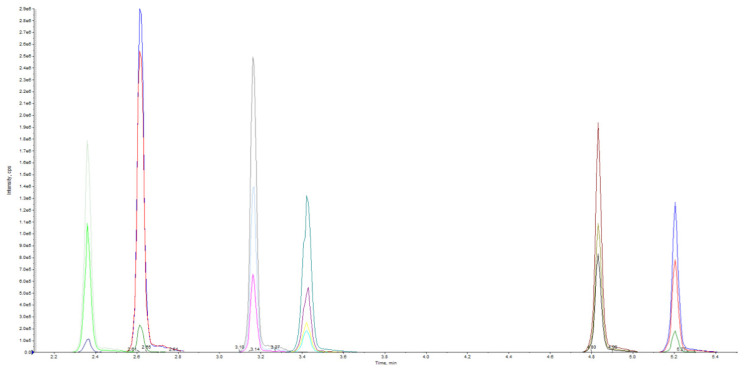
MRM extracted ion chromatogram of methanol standard solution.

The tuning parameters optimized in UHPLC-MS/MS to obtain fragment ions of high abundance for use in the multiple reaction monitoring (MRM) method are reported in Table 1 and Figure 2.

The identity of the observed fragment ions was confirmed by HRMS and collision-induced dissociation (CID) measurements in an Orbitrap comparing the theoretical to the experimental accurate masses, as reported in Table 2 (fragments of data are partially shown). The characteristic fragmentation patterns of URB compounds were subsequently interpreted as follows.

### 2.1. URB597

**Figure 3:**
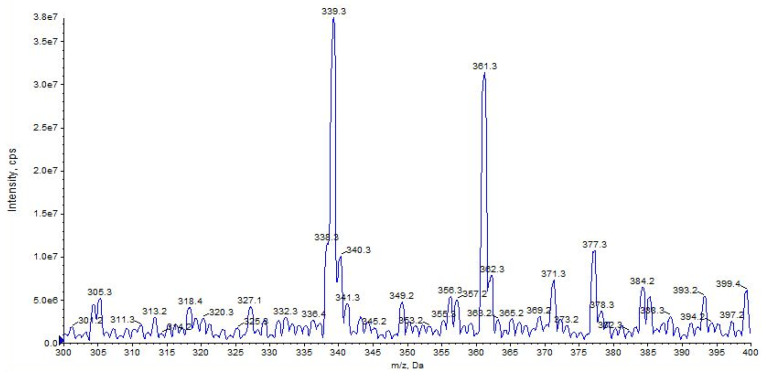
Representation of alkali metal peaks of URB597 compound. Exponential scale to base 10.

URB597 ([M + H]^+^ at *m*/*z* 339) shows a high affinity for the alkali metal with a high abundance of the sodium adduct [M + Na]^+^ at *m*/*z* 361, while a small amount of potassium adducts [M + K]^+^ at *m*/*z* 377 was detected (Figure 3).

**Figure 4:**
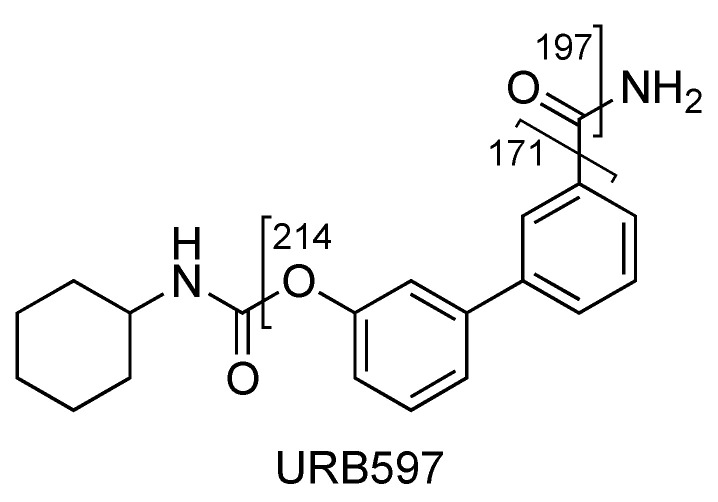
Representation of the collisional-induced fragment ions of URB597 compound.

**Figure 5:**
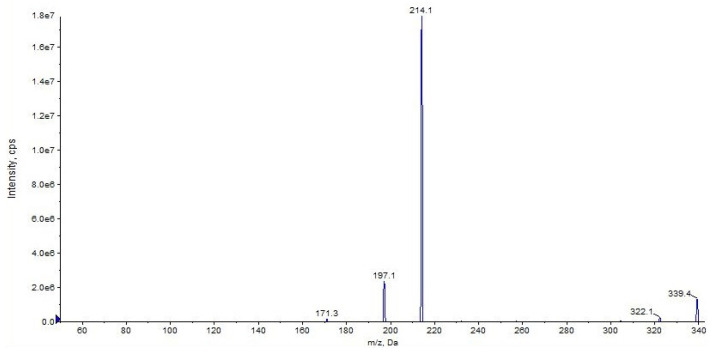
Product ion scan of URB597 compound obtained by QqQ. Exponential scale to base 10.

The fragment ion at *m*/*z* 214 is due to the loss of cyclohexyl isocyanate and that at *m*/*z* 197 is due to the loss of cyclohexyl isocyanate and ammonia (Figure 4 and Figure 5, CE 20 V). Other fragment ions are those at *m*/*z* 322 due to the loss of ammonia from 339 and at *m*/*z* 171, which differs from the fragment at *m*/*z* 214 due to the loss of the carbamoyl group (Figure 4 and Figure 5).

**Figure 6:**
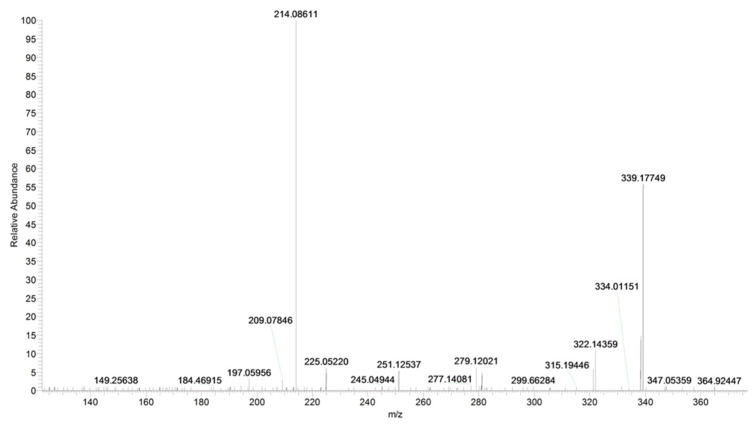
Product ion scan of URB597 compound obtained by HRMS.

The main fragmentation pathways are driven by α-cleavages to the carbonyl moieties (Table 2, Figure 6).

### 2.2. URB937

**Figure 7:**
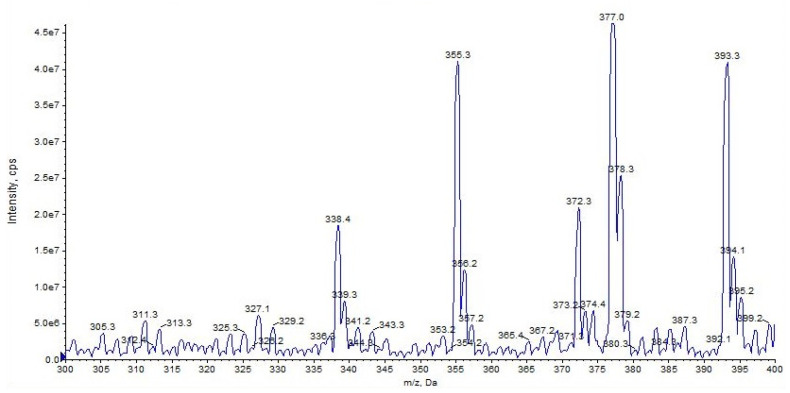
Representation of alkali metal peaks of URB937 compound.

URB937 differs from URB597 by the presence of a 6–hydroxy group on the proximal phenyl ring. URB937 ([M + H]^+^ *m*/*z* 355) shows the same fragmentation pattern as URB597, displaying fragment ions at *m*/*z* 377 [M + Na]^+^ and *m*/*z* 393 [M + K]^+^ but more affinity with alkali metal (Figure 7).

**Figure 8:**
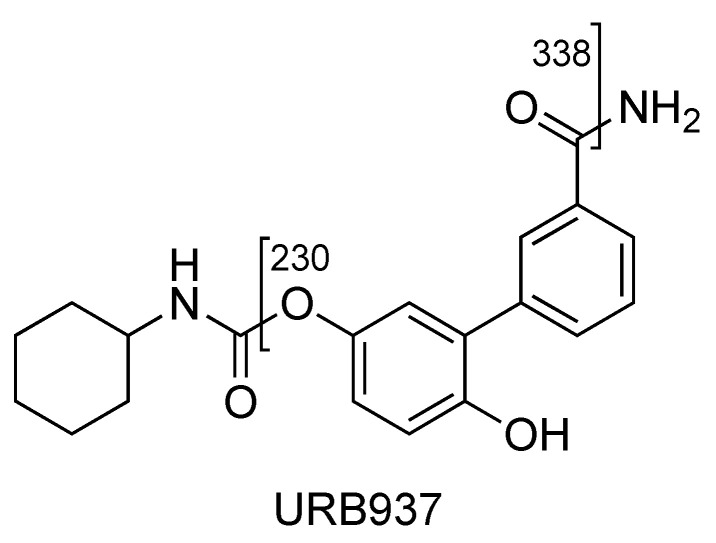
Representation of the collisional-induced fragment ions of URB937 compound.

The most abundant ion at *m*/*z* 230 results from the loss of cyclohexyl isocyanate (Figure 8, CE 20 V).

**Figure 9:**
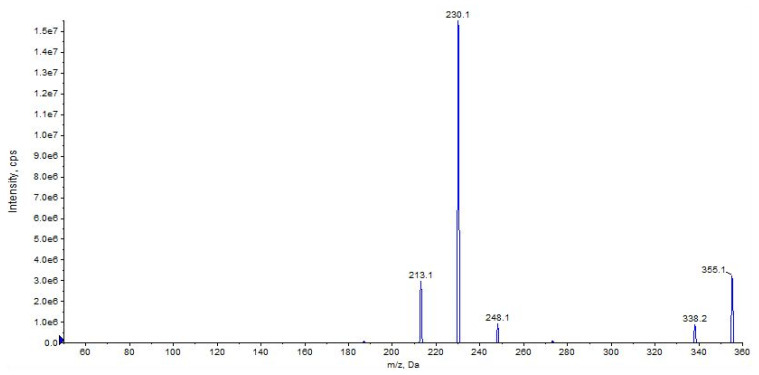
Product ion scan of URB937 compound obtained by QqQ. Exponential scale to base 10.

Other main characteristic fragments are observed at *m*/*z* 213 due to the loss of cyclohexyl isocyanate and ammonia, and *m*/*z* 338 due to the loss of ammonia only (Figure 8 and Figure 9).

**Figure 10:**
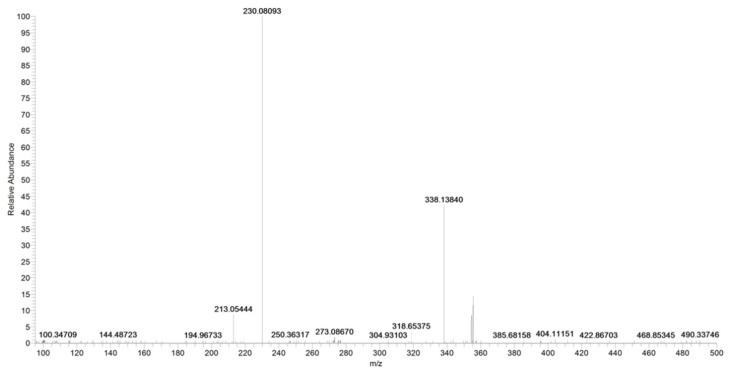
Product ion scan of URB937 compound obtained by HRMS.

The ion at *m*/*z* 187, due to the loss of the carbamoyl group, is observed only at higher collision energies (figure not shown). In the case of URB937, the presence of [M_2_ + Na] and [M_3_ + Na] at *m*/*z* 731.3057 and 1085.4636, respectively, was observed by accurate mass (Figure 10).

### 2.3. URB602

**Figure 11:**
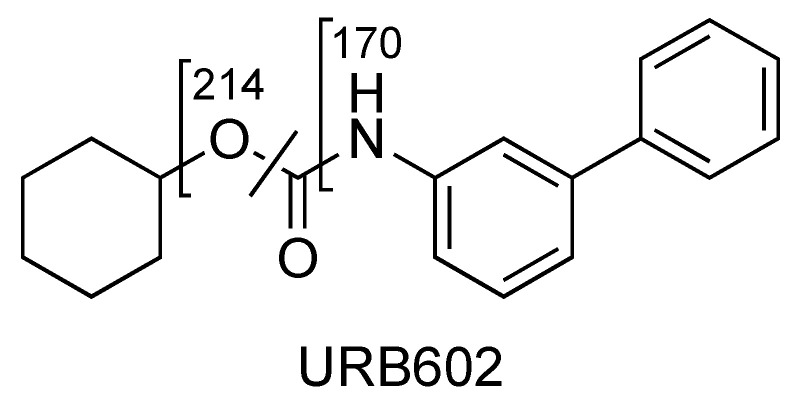
Representation of the collisional-induced fragment ions of URB602 compound.

**Figure 12:**
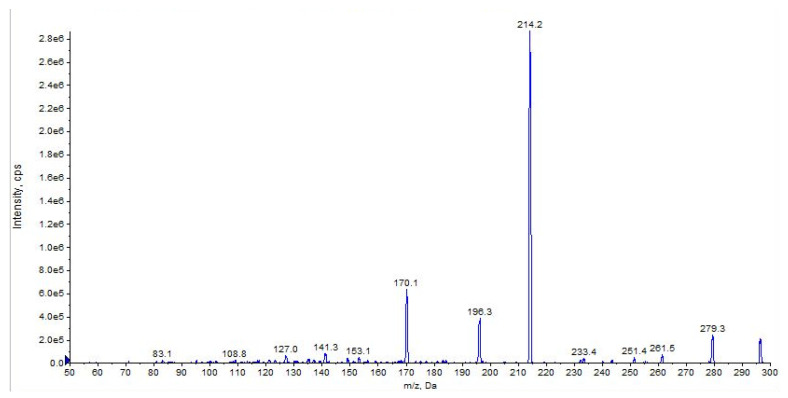
Product ion scan of URB602 compound obtained by QqQ. Exponential scale to base 10.

The main fragment ions of URB602 ([M + H]^+^ *m*/*z* 296) are those at *m*/*z* 214, due to the loss of cyclohexyl, at *m/z* 196 and *m/z* 170, depending on the losses of water and carbon anhydride, respectively (Figure 11 and Figure 12, CE 20 V).

**Figure 13:**
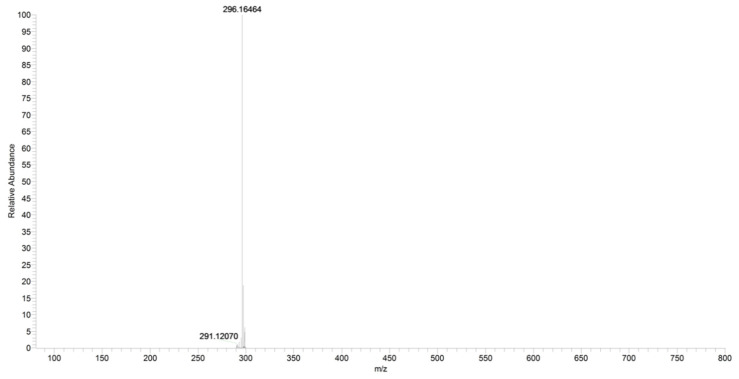
Scan of URB602 compound obtained by HRMS.

For URB602, a significant in-source fragmentation occurred after the direct injection of the URB602 solution (1 ppm). In fact, only a small amount of this protonated ion was observed in the scan mode in both QqQ and in HRMS (Figure 13). The poor ionization and different collisional conditions in the linear trap of Orbitrap did not permit an HRMS fragment ions characterization.

### 2.4. URB754

**Figure 14:**
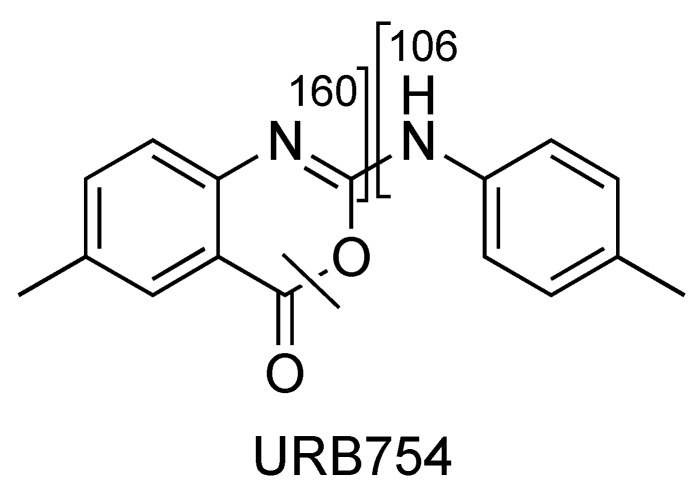
Representation of the collisional-induced fragment ions of URB754 compound.

**Figure 15:**
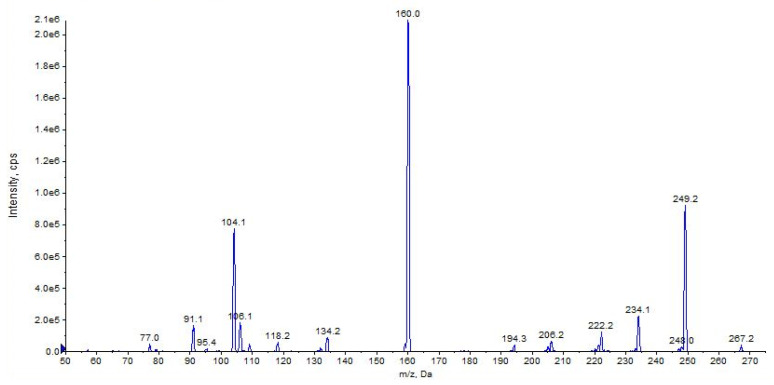
Product ion scan of URB754 compound obtained by QqQ. Exponential scale to base 10.

Characteristic MS/MS fragmentation patterns are shown in Figure 14 and Figure 15 (CE 40 V).

**Figure 16:**
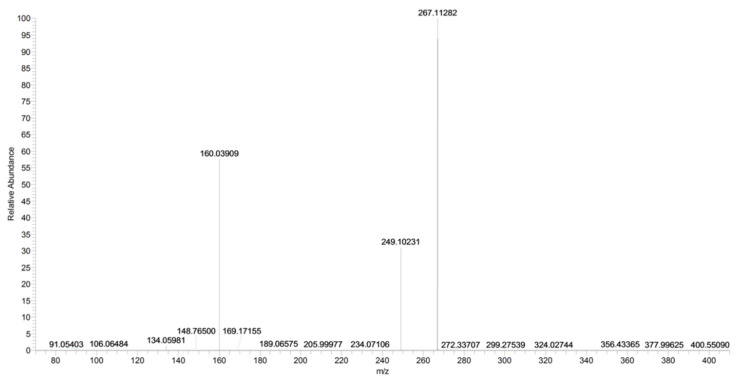
Product ion scan of URB754 compound obtained by HRMS.

The main fragment ions of URB754 ([M + H]^+^ at *m*/*z* 267) are 6-methyl-4-oxo-4*H*-3,1-benzoxazin-2-ylium at *m*/*z* 160, due to the loss of 4-methylaniline, and the ion at *m*/*z* 249, due to the loss of water caused by the opening of the 1,3-oxazin-6-one ring. A fragment ion at *m*/*z* 106 was also observed, caused by the formation of the toluidine derivative ion. The description of the accurate mass is shown in Figure 16.

### 2.5. URB447

**Figure 17:**
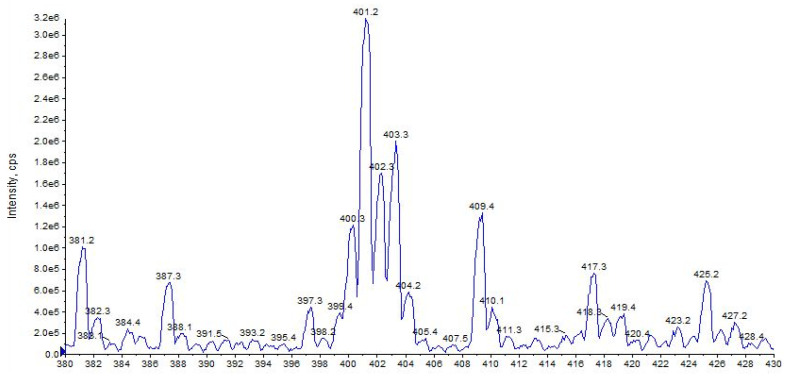
Representation of alkali metal peaks of URB447 compound. Exponential scale to base 10.

URB447 shows a quasi-molecular ion [M + H]^+^ at *m/z* 401 and 403, due to the presence of the characteristic isotopic pattern of chlorine ^35^Cl and ^37^Cl (Figure 17).

**Figure 18:**
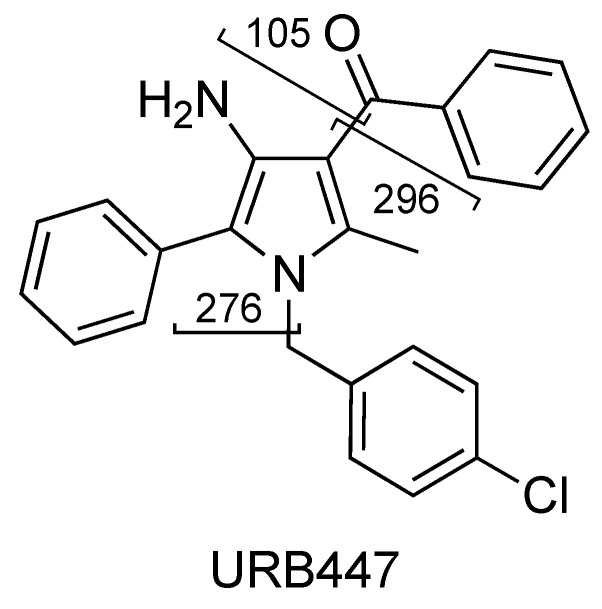
Representation of the collisional-induced fragment ions of URB447 compound.

**Figure 19:**
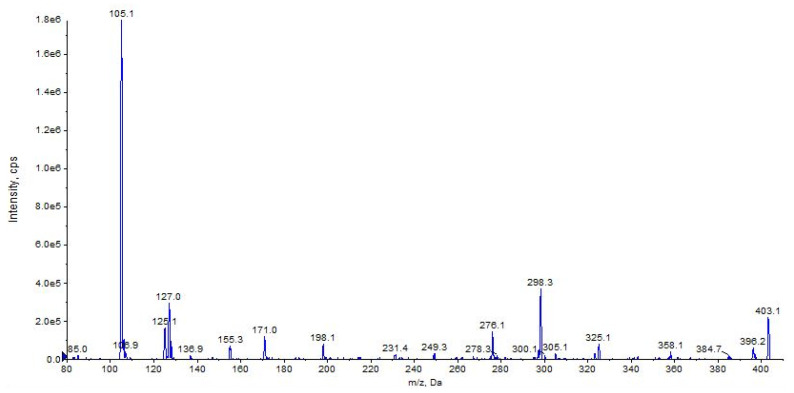
Product ion scan of URB447 compound obtained by QqQ. Exponential scale to base 10.

The fragment ions observed for URB447 are due to the cleavages of the carbon–carbon bond between the carbonyl and 3-pyrrolyl groups resulting in a benzoyl ion at *m/z* 105 or a 4-amino-1-[(4-chlorophenyl)methyl]-2-methyl-5-phenyl-1*H*-pyrrol-3-ylium ion at *m/z* 296 (Figure 18 and Figure 19, CE 35 V).

**Figure 20:**
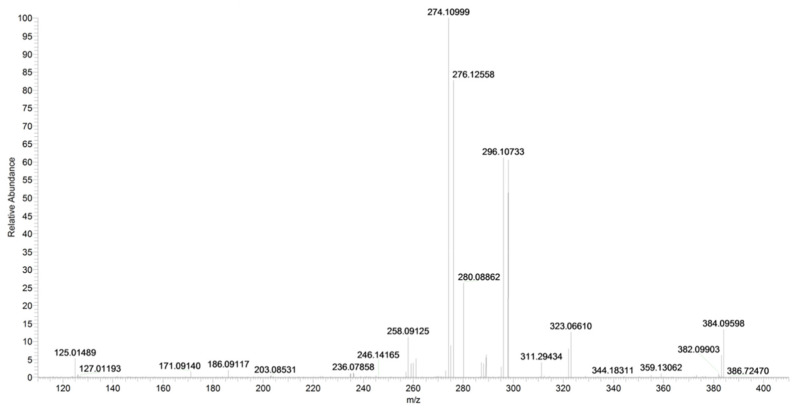
Product ion scan of URB447 compound obtained by HRMS.

A direct cleavage of the C–N bond between the 4-chlorobenzyl group and the *N*-pyrrolyl group results in a 4-chlorobenzylium ion at *m/z* 125 or 4-amino-3-benzoyl-2-methyl-5-phenyl-1*H*-pyrrol-1-ylium ion at *m*/*z* 276 (C_18_H_16_N_2_O) (Figure 18 and Figure 19). Analogue fragment ions at *m*/*z* 127 and at *m*/*z* 298 were observed starting from a similar precursor ion at *m/z* 403 (figure not shown). The description of the accurate mass is shown in Figure 20.

## 3. Materials and Methods

### 3.1. Standard and Solvents

JWH–018 (Internal Standard, IS) was supplied by Lipomed AG (Arlesheim, CH). Water, acetonitrile, isopropyl alcohol, and methanol LC-MS grade were furnished from PanReac AppliChem (Nova Chimica; Cinisello Balsamo, Italy). Formic acid LC-MS grade was provided by Sigma-Aldrich (Milan, Italy).

### 3.2. Chemicals

URB compounds were synthesized in the laboratories of the Department of Biomolecular Sciences of the University of Urbino Carlo Bo as described [41,42,43,44,45]. Information about the core assays is reported in [40,41,43,45,50]. Data regarding mp, NMR, and IR are present in [41,44,45,51,52]. Results on pharmacological tests are published in [40,41,42,43,45].

### 3.3. UHPLC-MS/MS

The characterization of compounds by UHPLC-MS/MS was carried out with a UHPLC system (Nexera X2, Shimazdu, Kyoto, Japan) coupled with a triple quadrupole mass spectrometer (Hybrid System 4000 Qtrap, AB Sciex, Framingham, MA, USA). Chromatographic separation was performed on a 50 × 2.1 mm KINETEX EVO 1.9 μm C18 column (Phenomenex, Bologna, Italy). The mobile phase was composed of solvent “A” (0.1% formic acid) and “B” (0.1% formic acid in acetonitrile with 5% water). The initial chromatographic conditions were 20% “B”, and the gradient was programmed as follows: 0–0.1 min hold 20% “B”, 0.1–6.0 min to 80% “B”, and 6.0–6.1 min to 100% “B”, and it was kept for 3 min. Finally, the initial condition was restored and kept for 2 min to equilibrate the system. Total run time was 11 min at the constant flow rate (0.5 mL/min); the column temperature was set at 40 °C; the autosampler was maintained at 10 °C. The mass spectrometer operated in positive ion mode using ESI TurboIonSpray^®^ under the following conditions: curtain gas (CUR) 30.0 psi, ion spray voltage (IS) 4000 V, temperature (TEM) 500 °C, ion source gas 1 (GS1) 40.0 psi, and ion GS2 55.0 psi. Identification was performed using MRM mode. MRM transitions were experimentally established. At least three MRM transitions were monitored for each compound. A total of 19 transition were monitored with an MRM pause time of 5 ms and a dwell time of 20 ms for each transition; the total scan time was 0.47 s.

### 3.4. HRMS Compound Characterization

The methanolic solutions of the individual standards (URB597, URB937, URB602, URB754, and URB447) were prepared separately in 10 mL volumetric flasks at an approximate concentration of 100 or 1000 μg/mL. Each drug was diluted to a concentration from 0.1 to 1 ppm in methanol. The determination of the exact mass of ionic species in the URB compounds was performed on HRMS experiments using a Linear Trap Quadrupole (LTQ) Orbitrap XL hybrid mass spectrometry (Thermo Scientific^TM^, ThermoFisher Scientific, Waltham, MA, USA) equipped with an electrospray ion source operated in positive ion mode. Direct infusion of compounds into the MS ion source was performed using a syringe pump at a flow rate of 10 µL/min. The protonated molecules of each compound were isolated in the linear ion trap and fragmented at varying collision energies in order to identify the most abundant and characteristic product ions.

## 4. Conclusions

The present study integrates and extends the mass spectrometry characterization of synthetic drugs that are able to interact with the cannabinoid system. It provides the first description of URB937, URB602, URB754, and URB447 compounds by CID in tandem with the quadrupole mass spectrometry and hybrid ion trap-orbitrap mass spectrometry. It should also be considered that, so far, the analyses and characterizations have been carried out in MS (EI) [44,45,51,52] or MS (ESI) [41] only. The methods used here have made it possible to unequivocally characterize the molecules under examination with the aim to determine the operating conditions for their accurate identification and, therefore, laying the foundations for the future development of analytical methods aimed at the early screening of NPS in seizures or biological samples. However, these substances have long been monitored as potential alerts [48,53]. In this respect, studies on the metabolism of the URB compounds are necessary, and the work is in progress.

## Figures and Tables

**Table 1:** Compound-dependent parameters for optimal monitoring of multiple reactions.

Compound (Elementary Formula)	RT	[M + H]^+^ (*m*/*z*)	Product Ions (*m*/*z*)	QTT/QLT Ion Ratio (%)	DP (V)	EP (V)	CE (V)	CXP (V)
JWH-018 (C_24_H_23_NO) (internal standard)	5.22	342	155	QTT	110	10	35	11
			127	60	110	10	70	6
			145	33	110	10	59	11
URB597 (C_20_H_22_N_2_O_3_)	3.16	339	214	QTT	78	10	18	11
			197	62	78	10	31	17
			171	30	78	10	39	12
URB937	2.62	355	338	6	80	10	16	8
(C_20_H_22_N_2_O_4_)			230	QTT	80	10	22	5
			213	90	80	10	32	11
URB602 (C_19_H_21_NO_2_)	4.84	296	214	QTT	45	10	14	10
			196	50	45	10	29	15
			170	67	45	10	28	15
URB754 (C_16_H_14_N_2_O_2_)	2.36	267	249	63	70	10	34	18
			160	QTT	70	10	32	11
			106	6	70	10	40	7
URB447 (C_25_H_21_ClN_2_O)	3.43	401	296	45	90	10	34	7
			276	25	90	10	31	6
			105	QTT	90	10	38	5
		403	298	14	90	10	32	7

RT: Retention time, QTT: Quantifying Transition, QLT: Qualifying Transition, DP: Declustering Potential, EP: Entrance Potential, CE: Collision Energy, and CXP: Collision Cell Exit Potential.

**Table 2:** HRMS of protonated molecules and CID-induced fragment ions.

Compound	Elementary Formula (MH^+^)	Observed *m/z*	Theoretical *m/z*	Δppm
URB597	C_20_H_23_N_2_O_3_^+^	339.1703	339.1703	–
	C_13_H_12_NO_2_^+^	214.0861	214.0862	−0.5
	C_13_H_9_O_2_^+^	197.0596	197.0597	−0.5
	C_12_H_11_O^+^	171.0801	171.0804	−1.7
URB937	C_20_H_20_NO_4_Na^+^	377.1474	377.1471	0.8
	C_20_H_20_NO_4_^+^	338.1384	338.1387	−0.9
	C_13_H_12_NO_3_^+^	230.0809	230.0812	−1.3
	C_13_H_9_O_3_^+^	213.0544	213.0546	−0.9
URB602	C_19_H_22_NO_2_^+^	296.1646	296.1650	−1.3
URB754	C_16_H_15_N_2_O_2_^+^	267.1128	267.1128	–
	C_16_H_13_N_2_O^+^	249.1023	249.1022	0.4
	C_9_H_6_NO_2_^+^	160.0391	160.0393	−1.2
	C_7_H_8_N^+^	106.0648	106.0651	−2.8
URB447	C_25_H_22_ClN_2_O^+^	401.1413	401.1415	−0.5
	C_18_H_17_N_2_Cl^+^	296.1073	296.1074	0.3
	C_18_H_16_N_2_O^+^	276.1256	276.1257	−0.4

## Data Availability

Data is contained within the article.

## References

[1] Kumar P., Mahato D.K., Kamle M., Borah R., Sharma B., Pandhi S., Tripathi V., Yadav H.S., Devi S., Patil U. (2021). Pharmacological properties, therapeutic potential, and legal status of *Cannabis sativa* L.: An overview. Phytother. Res..

[2] Pagano C., Navarra G., Coppola L., Avilia G., Bifulco M., Laezza C. (2022). Cannabinoids: Therapeutic use in clinical practice. Int. J. Mol. Sci..

[3] Gülck T., Møller B.L. (2020). Phytocannabinoids: Origins and biosynthesis. Trends Plant Sci..

[4] (2022). European Monitoring Centre for Drugs and Drug Addiction. European Drug Reports. Trends and Developments. https://www.emcdda.europa.eu/system/files/publications/14644/TDAT22001ENN.pdf.

[5] Devane W.A., Dysarz F.A., Johnson M.R., Melvin L.S., Howlett A.C. (1988). Determination and characterization of a cannabinoid receptor in rat brain. Mol. Pharmacol..

[6] Matsuda L.A., Lolait S.J., Brownstein M.J., Young A.C., Bonner T.I. (1990). Structure of a cannabinoid receptor and functional ex-pression of the cloned cDNA. Nature.

[7] Munro S., Thomas K.L., Abu-Shaar M. (1993). Molecular characterization of a peripheral receptor for cannabinoids. Nature.

[8] Lutz B. (2020). Neurobiology of cannabinoid receptor signaling. Dialogues Clin. Neurosci..

[9] Shahbazi F., Grandi V., Banerjee A., Trant J.F. (2020). Cannabinoids and cannabinoid receptors: The story so far. iScience.

[10] Ren S., Wang Z., Zhang Y., Chen N. (2020). Potential application of endocannabinoid system agents in neuropsychiatric and neuro-degenerative diseases—Focusing on FAAH/MAGL inhibitors. Acta Pharmacol. Sin..

[11] Van Sickle M.D., Duncan M., Kingsley P.J., Mouihate A., Urbani P., Mackie K., Stella N., Makriyannis A., Piomelli D., Davison J.S. (2005). Identification and functional characterization of brainstem cannabinoid CB2 receptors. Science.

[12] Tang X., Liu Z., Li X., Wang J., Li L. (2021). Cannabinoid receptors in myocardial injury: A brother born to rival. Int. J. Mol. Sci..

[13] Devane W.A., Hanus L., Breuer A., Pertwee R.G., Stevenson L.A., Griffin G., Gibson D., Mandelbaum A., Etinger A., Mech-oulam R. (1992). Isolation and structure of a brain constituent that binds to the cannabinoid receptor. Science.

[14] Mechoulam R., Ben-Shabat S., Hanus L., Ligumsky M., Kaminski N.E., Schatz A.R., Gopher A., Almog S., Martin B.R., Compton D.R. (1995). Identification of an endogenous 2-monoglyceride, present in canine gut, that binds to cannabinoid receptors. Biochem. Pharmacol..

[15] Sugiura T., Kondo S., Sukagawa A., Nakane S., Shinoda A., Itoh K., Yamashita A., Waku K. (1995). 2-Arachidonoylglycerol: A possible endogenous cannabinoid receptor ligand in brain. Biochem. Biophys. Res. Commun..

[16] Piomelli D. (2003). The molecular logic of endocannabinoid signalling. Nat. Rev. Neurosci..

[17] Augustin S.M., Lovinger D.M. (2018). Functional Relevance of endocannabinoid-dependent synaptic plasticity in the central nervous system. ACS Chem. Neurosci..

[18] Lowe H., Toyang N., Steele B., Bryant J., Ngwa W. (2021). The endocannabinoid system: A potential target for the treatment of various diseases. Int. J. Mol. Sci..

[19] Piomelli D., Mabou Tagne A. (2022). Endocannabinoid-based therapies. Ann. Rev. Pharmacol. Toxicol..

[20] Gaoni Y., Mechoulam R. (1964). Isolation, structure, and partial synthesis of an active constituent of hashish. J. Am. Chem. Soc..

[21] Peng J., Fan M., An C., Ni F., Huang W., Luo J. (2022). A narrative review of molecular mechanism and therapeutic effect of cannabidiol (CBD). Basic Clin. Pharmacol. Toxicol..

[22] Manera C., Bertini S. (2021). Cannabinoid-Based Medicines and Multiple Sclerosis. Adv. Exp. Med. Biol..

[23] Jazz Pharmaceutics Website. https://www.jazzpharma.com/medicines/our-medicines/.

[24] Pertwee R.G. (2012). Targeting the endocannabinoid system with cannabinoid receptor agonists: Pharmacological strategies and therapeutic possibilities. Phil. Trans. R. Soc. B.

[25] De Luca M.A., Fattore L. (2018). Therapeutic use of synthetic cannabinoids: Still an open issue?. Clin. Ther..

[26] An D., Peigneur S., Hendrickx L.A., Tytgat J. (2020). Targeting cannabinoid receptors: Current status and prospects of natural prod-ucts. Int. J. Mol. Sci..

[27] Estrada J.A., Contreras I. (2020). Endocannabinoid receptors in the CNS: Potential drug targets for the prevention and treatment of neurologic and psychiatric disorders. Curr. Neuropharmacol..

[28] Wiley J.L., Compton D.R., Dai D., Lainton J.A.H., Phillips M., Huffman J.W., Martin B.R. (1998). Structure-activity relationships of indole- and pyrrole-derived cannabinoids. J. Pharmacol. Exp. Ther..

[29] Auwärter V., Dresen S., Weinmann W., Müller M., Pütz M., Ferreirós N. (2009). ‘Spice’ and other herbal blends: Harmless incense or cannabinoid designer drugs?. J. Mass Spectrom..

[30] Atwood B.K., Huffman J., Straiker A., Mackie K. (2010). JWH018, a common constituent of ‘Spice’ herbal blends, is a potent and efficacious cannabinoid CB receptor agonist. Br. J. Pharmacol..

[31] UNODC Early Warning Advisory Toxicology Highlights Current NPS Threats, Volume II, January 2020. https://www.unodc.org/documents/scientific/Current_NPS_Threats_Volume_II_Web.pdf.

[32] Shafi A., Berry A.J., Sumnall H., Wood D.M., Tracy D.K. (2020). New psychoactive substances: A review and updates. Ther. Adv. Psychopharmacol..

[33] Giorgetti A., Pascali J.P., Fai P., Pelletti G., Gabbin A., Franchetti G., Cecchetto G., Viel G. (2021). Molecular mechanisms of action of novel psychoactive substances (NPS). A new threat for young drug users with forensic-toxicological implications. Life.

[34] UNODC Early Warning Advisory Toxicology Highlights (2021). Current NPS Threats, Volume IV. https://www.unodc.org/documents/scientific/NPS_threats-IV.pdf.

[35] Chung E.Y., Cha H.J., Min H.K., Yun J. (2021). Pharmacology and adverse effects of new psychoactive substances: Synthetic cannabinoid receptor agonists. Arch. Pharm. Res..

[36] UNODC Early Warning Advisory on New Psychoactive Substances (2021). What Are NPS?. https://www.unodc.org/LSS/Page/NPS.

[37] European Monitoring Centre for Drugs and Drug Addiction (2021). Synthetic Cannabinoids in Europe—A Review. https://www.emcdda.europa.eu/system/files/publications/14035/Synthetic-cannabinoids-in-Europe-EMCDDA-technical-report.pdf.

[38] Markham J., Sparkes E., Boyd R., Chen S., Manning J.J., Finlay D., Lai F., McGregor E., Maloney C.J., Gerona R.R. (2022). Defining steric requirements at CB1 and CB2 cannabinoid receptors using synthetic cannabinoid receptor agonists 5F-AB-PINACA, 5F-ADB-PINACA, PX-1, PX-2, NNL-1, and their analogues. ACS Chem. Neurosci..

[39] Alves V.L., Gonçalves J.L., Aguiar J., Teixeira H.M., Câmara J.S. (2020). The synthetic cannabinoids phenomenon: From structure to toxicological properties. A review. Crit. Rev. Toxicol..

[40] Kathuria S., Gaetani S., Fegley D., Valiño F., Duranti A., Tontini A., Mor M., Tarzia G., La Rana G., Calignano A. (2003). Modulation of anxiety through blockade of anandamide hydrolysis. Nat. Med..

[41] Clapper J.R., Moreno-Sanz G., Russo R., Guijarro A., Vacondio F., Duranti A., Tontini A., Sanchini S., Sciolino N.R., Spradley J.M. (2010). Anandamide suppresses pain initiation through a peripheral endocannabinoid mechanism. Nat. Neurosci..

[42] Hohmann A.G., Suplita R.L., Bolton N.M., Neely M.H., Fegley D., Mangieri R., Krey J.F., Walker J.M., Holmes P.V., Crystal J.D. (2005). An endocannabinoid mechanism for stress-induced analgesia. Nature.

[43] Makara J.K., Mor M., Fegley D., Szabó S.I., Kathuria S., Astarita G., Duranti A., Tontini A., Tarzia G., Rivara S. (2005). Selective inhibition of 2-AG hydrolysis enhances endocannabinoid signaling in hippocampus. Nat. Neurosci..

[44] Tarzia G., Antonietti F., Duranti A., Tontini A., Mor M., Rivara S., Traldi P., Astarita G., King A., Clapper J.R. (2007). Identification of a bioactive impurity in a commercial sample of 6-methyl-2-*p*-tolylaminobenzo[*d*][1,3]oxazin-4-one (URB754). Ann. Chim..

[45] LoVerme J., Duranti A., Tontini A., Spadoni G., Mor M., Rivara S., Stella N., Xu C., Tarzia G., Piomelli D. (2009). Synthesis and characterization of a peripherally restricted CB_1_ cannabinoid antagonist, URB447, that reduces feeding and body-weight gain in mice. Bioorg. Med. Chem. Lett..

[46] Benedicto A., Arteta B., Duranti A., Alonso-Alconada D. (2022). The synthetic cannabinoid URB447 exerts antitumor and antimetastatic effect in melanoma and colon cancer. Pharmaceuticals.

[47] Nakajima J., Takahashi M., Seto T., Kanai C., Suzuki J., Yoshida M., Uemura N., Hamano T. (2013). Analysis of azepane isomers of AM-2233 and AM-1220, and detection of an inhibitor of fatty acid amide hydrolase [3′-(aminocarbonyl)(1,1′-biphenyl)-3-yl]-cyclohexylcarbamate (URB597) obtained as designer drugs in the Tokyo area. Forensic Toxicol..

[48] Shanks K.G., Behonick G.S., Dahn T., Terrell A. (2013). Identification of novel third-generation synthetic cannabinoids in products by ultra-performance liquid chromatography and time-of-flight mass spectrometry. J. Anal. Toxicol..

[49] http://www.emcdda.europa.eu/attachements.cfm/att_229598_EN_TDAN14001ENN.pdf.

[50] Uchiyama N., Kawamura M., Kikura-Hanajiri R., Goda Y. (2013). URB-754: A new class of designer drug and 12 synthetic cannabinoids detected in illegal products. Forensic Sci. Int..

[51] Tarzia G., Duranti A., Tontini A., Piersanti G., Mor M., Rivara S., Plazzi P.V., Park C., Kathuria S., Piomelli D. (2003). Design, synthesis, and structure–activity relationships of alkylcarbamic acid aryl esters, a new class of fatty acid amide hydrolase inhibitors. J. Med. Chem..

[52] Mor M., Rivara S., Lodola A., Plazzi P.V., Tarzia G., Duranti A., Tontini A., Piersanti G., Kathuria S., Piomelli D. (2004). Cyclohexylcarbamic Acid 3′- or 4′-substituted biphenyl-3-yl esters as fatty acid amide hydrolase inhibitors: Synthesis, quantitative structure-activity relationships, and molecular modelling studies. J. Med. Chem..

[53] https://namsdl.org/wp-content/uploads/Summary-of-Synthetic-Drugs-Bills-and-Proposed-Regulations.pdf.

